# Risk Factors for Readmission in Heart Failure Within 90 Days

**DOI:** 10.7759/cureus.50236

**Published:** 2023-12-09

**Authors:** Alhassin S Alsulymani, Waddah Ashram, Abdullah Alghamdi, Hatoon W Hafiz, Ahmed M Ghunaim, Basel Aljehani, Ahmed Aljabri, Ghadi Alzahrani

**Affiliations:** 1 Medicine, King Abdulaziz University Faculty of Medicine, Jeddah, SAU; 2 Internal Medicine, King Abdulaziz University Hospital, Jeddah, SAU; 3 Infectious Diseases, King Abdulaziz University Faculty of Medicine, Jeddah, SAU; 4 Internal Medicine, King Abdulaziz University Faculty of Medicine, Jeddah, SAU; 5 Pathology, King Abdulaziz University Faculty of Medicine, Jeddah, SAU

**Keywords:** hypertension, drug compliance, acute decompensated heart failure, readmission risk, heart failure

## Abstract

Background: Hospital readmission rates among heart failure (HF) patients remain a persistent challenge. While various risk factors have been identified, their impact on individual patients varies, and not all patients with these risk factors will necessarily experience readmission within 90 days. This study aimed to identify and assess the significance of risk factors associated with readmission of HF patients within 90 days.

Methods: A retrospective cohort study was conducted at King Abdulaziz University Hospital in Jeddah, Saudi Arabia. The sample size comprised 130 patients. Data was collected from hospital records of all HF patients readmitted within 90 days of discharge between 2018 and 2022.

Results: The study included 130 patients. The majority (70%) were between 51 and 70 years old. Most patients had a hospital stay ranging from 1 to 10 days (83.1%). Shortness of breath (SOB) was the most common reason for readmission, accounting for 80% of cases. Other reasons included chest pain (CP) (6.2%), orthopnea (13.1%), paroxysmal nocturnal dyspnea (PND) (5.4%), lower limb edema (LLE) issues (1.5%), and heart failure (HF) itself (2.3%). Less common reasons included fever (1.5%), pneumonia (1.5%), altered level of consciousness (1.5%), and urinary tract infection (1.5%).

Conclusion: Readmission rates for HF patients remain high, attributed to factors such as non-adherence to medication and lifestyle changes, comorbidities, inadequate discharge planning, and social determinants of health. Males may be more prone to experiencing SOB and subsequently requiring readmission at a higher rate. These findings underscore the need for comprehensive interventions that address these risk factors to minimize readmission rates and improve patient outcomes.

## Introduction

Heart failure (HF) is a chronic condition characterized by the inability of the heart to pump sufficient blood to meet the body's needs [1]. It is a leading cause of hospital admissions worldwide [2].

Currently, an estimated 1% to 2% of adults are affected by HF. In Saudi Arabia, approximately 37,935 new cases of HF are diagnosed annually, with a projected prevalence of 455,222 patients [3]. According to the Healthcare Cost and Utilization Project, an estimated 134,500 HF patients required readmission in 2011, at a staggering cost of $1.7 billion [3].

Readmission rates for HF patients within 30 days of discharge can be as high as 24.8% in the United States. Two additional studies [4-6] conducted in the USA reported readmission rates of 23% and 21.2%, respectively, within 30 and 90 days.

Several risk factors are associated with readmission in HF patients. These factors can be categorized into two main groups: medical and demographic factors [2,6,7-9]. Medical factors include age, gender, body mass index (BMI), medication adherence, smoking, history of prior hospital admissions, length of hospital stay, and the presence of comorbidities such as diabetes mellitus, renal failure, coronary artery disease, and lung disease [2,6,7-9].

Despite numerous studies investigating risk factors for readmission in HF patients within a 90-day period, few have compared readmission rates across different risk factors, and none have been conducted in Jeddah. This study aims to identify and assess the significance of risk factors associated with readmission of HF patients within 90 days.

## Materials and methods

This retrospective cohort study was conducted during 2023 from January to September at King Abdulaziz University Hospital (KAUH), a tertiary center in Jeddah, Saudi Arabia. It was conducted in the Department of Medicine and approved by the ethics committee of KAUH. A comprehensive review was undertaken on the medical records of 1592 adult patients from January 2018 to December 2022. These individuals were identified with HF by the presence of the International Classification of Disease (ICD)-10 Code I50.9. They were diagnosed with HF based on an echocardiogram and sought medical attention across multiple departments within our esteemed hospital, of whom 130 were readmitted in 90 days since their last admission. The sample size was calculated using a margin of error of 6% and a confidence interval of 95%. 

Information collected from medical records included age, gender, reason for readmission, length of stay in hospital, BMI, medication compliance, alcohol, other diagnoses besides HF, time and type of discharge, and name of apartment admitted. A checklist was employed with the purpose of extracting pertinent information from the hospital records. This comprehensive data sheet obtained by using Microsoft Excel also encompassed inquiries regarding demographic data (except for patient names), instances of readmission subsequent to HF, risk factors associated with readmission, and the duration of each hospital admission. The relationship between these risk factors and the readmission of HF patients within 90 days was considered the primary outcome of the study. 

The research study encompassed a cohort of individuals representing both genders who surpassed the age threshold of 16 and were identified as having received a clinical diagnosis of HF. In order to maintain the study's focus on HF specifically, patients with other coexisting heart diseases were excluded. These stringent criteria were implemented to ensure a homogenous sample that would enable a more focused investigation into the variables related to HF in the designated patient group. 

Statistical analysis was conducted using Statistical Package for Social Sciences (SPSS), version 21.0 (IBM Corp. Armonk, NY), where continuous variables were described using mean and standard deviation, while categorical variables were presented as numbers and percentages. Categorical variables were subjected to univariate analysis using the chi-square test. Logistic regression was performed to assess associations between variables. Odds ratios, confidence intervals for odds ratios, and p-values for statistically significant associations were generated. Prevalence data was presented as percentages with 95% confidence intervals. In this study, p-values less than 0.05 were considered statistically significant. Patient confidentiality and data privacy were of utmost importance, and steps were taken to ensure these aspects were prioritized. There were no ethical issues arising from the study, as participant names were not used.

## Results

Sociodemographic characteristics of participants

The purpose of the present study was to assess the significance of risk factors associated with the readmission of HF patients within 90 days. The sample size included 130 patients, recruited from King Abdulaziz University Hospital.

As shown in Table 1, the sociodemographic characteristics of participants, 79 (60.8%) of participants were males and 51 (39.2%) were females. The majority of the participants, 72 (66.2%), were between 51-70 years of age. Among these, 38 (29.2%) were between the ages of 61 and 70, and 34 (26.2%) were between the ages of 51 and 60. Thirty-nine (30%) of the patients were between the ages of 71 and 90, with the age group of 71 to 80 having the largest percentage 20 (15.4%). A lesser percentage of patients, 4 (3.1%) and 15 (11.5%), respectively, were between the ages of 30 to 40 and 41 to 50. 

**Table 1:** Sociodemographic characteristics of participants (n=130). This table provides information on the sociodemographic characteristics of participants in the study population. The data is presented in terms of the absolute number of individuals affected and the corresponding percentage of the total population.

Parameter		No.	Percent
Age	30 - 40	4	3.1
	41 - 50	15	11.5
	51- 60	34	26.2
	61 - 70	38	29.2
	71- 80	20	15.4
	81 - 90	19	14.6
Gender	Male	79	60.8
	Female	51	39.2
Length of hospital stay (days)​​​​​	1 -10	108	83.1
	11 - 20	19	14.6
	More than 20	3	2.3
BMI​​​​	Underweight (Below 18.5)	3	2.3
	Normal (18.5 - 24.9)	33	25.4
	Overweight (25 - 29.9)	37	28.5
	Obese (30 or more)	57	43.8

The bulk of patients, 108 (83.1%), had a hospital stay ranging from 1 to 10 days (Table 1). A smaller percentage 19 (14.6%) had a stay of 11 to 20 days, and only 3 (2.3%) had a stay of more than 20 days (Table 1). This implies that most patients had relatively short hospital stays (Table 1). 

Regarding patient BMI categories, it revealed that the majority 57 (43.8%) were classified as obese (BMI of 30 or more) (Table 1). The remaining patients were divided between the categories of normal weight 33 (25.4%) and overweight 37 (28.5%) (Table 1). and only 3 (2.3%) were underweight (BMI below 18.5) (Table 1). This suggests that a higher percentage of patients in the dataset are obese (Table 1). 

These findings shed light on the clinical and demographic traits of the patient population that were part of this study and provide insight into the profile of HF patients in this setting (Table 1). 

Medical conditions and prevalence

Table 2 provides information on medication compliance, medical conditions, and substance abuse among the study participants. The data is presented in terms of the absolute number of individuals affected and the corresponding percentage of the total population. 

**Table 2:** Medical conditions as risk factors for readmission among study participants (n=130). This table provides information on medication compliance, medical conditions, and substance abuse among the study participants. The data is presented in terms of the absolute number of individuals affected and the corresponding percentage of the total population.

Parameter		No.	Percent
Medication compliance	Yes	60	46.2
	No	31	23.8
	Unknown	39	30.0
Alcohol	Yes	1	.8
	No	129	99.2
AIDS	No	130	100.0
Arrhythmias	Yes	35	26.9
	No	95	73.1
Chronic pulmonary disease	Yes	35	26.9
	No	95	73.1
Chronic kidney disease	Yes	45	34.6
	No	85	65.4
Depression	Yes	3	2.3
	No	127	97.7
Drug abuse	Yes	2	1.5
	No	128	98.5
Hypertension	Yes (not on medication)	6	4.6
	Yes (on medication)	108	83.1
	No	16	12.3
Hypothyroidism	Yes	19	14.6
	No	111	85.4
Liver disease	Yes	8	6.2
	No	122	93.8
Lymphoma	No	130	100.0
Metastatic cancer	Yes	1	.8
	No	129	99.2
Obesity	Yes	58	44.6
	No	72	55.4
Neurological disorder	Yes	12	9.2
	No	118	90.8
Obstructive sleep apnea	Yes	34	26.2
	No	96	73.8
Paralysis	No	130	100.0
Peptic ulcer disease	Yes	1	.8
	No	129	99.2
Peripheral vascular disease	Yes	7	5.4
	No	123	94.6
Psychoses	Yes	2	1.5
	No	128	98.5
Pulmonary circulation disorder	Yes	13	10.0
	No	117	90.0
Rheumatoid arthritis	Yes	2	1.5
	No	128	98.5
Renal failure	Yes	15	11.5
	No	115	88.5
Solid tumor without metastasis	Yes	9	6.9
	No	121	93.1
Valvular disease	Yes	10	7.7
	No	120	92.3
Weight loss	Yes	11	8.5
	No	119	91.5
Type 1 Diabetes	No	118	90.8
	Yes (controlled)	1	.8
	Yes (uncontrolled or unknown)	11	8.5
Type 2 Diabetes	Yes (controlled)	24	18.5
	Yes (uncontrolled or unknown)	59	45.4
	No	47	36.2
Smoker	Yes	14	10.8
	No	103	79.2
	Ex	13	10.0
Anaemia	Yes	95	73.1
	No	35	26.9
Coagulopathy	Yes	47	36.2
	No	83	63.8
Dyslipidemia	Yes	32	24.6
	No	98	75.4
Fluid and electrolyte disorder	Yes	71	54.6
	No	59	45.4

Medication compliance: Regarding medication compliance, 60 (46.2%) of participants reported consistent adherence to their prescribed medications, while 31 (23.8%) admitted to non-compliance (Table 2). The compliance status of the remaining 39 (30.0%) was unknown (Table 2). 

Medical conditions: The most prevalent medical conditions were anemia at 95 (73.1%), fluid and electrolyte disorder at 71 (54.6%), and hypertension was further categorized into individuals on medication and those not on medication (Table 2). Among the participants, 6 (4.6%) were not on medication for hypertension, while 108 (83.1%) were receiving treatment (Table 2). Type 2 diabetes was reported by 24 (18.5%) of patients, with controlled diabetes in 14 (18.5%) and uncontrolled or unknown status in 59 (45.4%) (Table 2). In the case of type 1 diabetes, 1 (0.8%) had controlled type 1 diabetes and 11 (8.5%) had uncontrolled or unknown status of type 1 diabetes (Table 2). In addition, obesity at 58 (44.6%), coagulopathy at 47 (36.2%), and chronic kidney disease at 45 (34.6%) were also common among the participants (Table 2). Arrhythmias were present in 35 (26.9%) of participants. Similarly, chronic pulmonary disease affected 35 (26.9%) of participants. Moreover, there were reports of other medical conditions such as obstructive sleep apnea at 34 (26.2%), dyslipidemia at 32 (24.6%), hypothyroidism at 19 (14.6%), renal failure at 15 (11.5%), pulmonary circulation disorder at 13 (10.0%), and neurological disorders at 12 (9.2%). Furthermore, weight loss, valvular disease, solid tumor without metastasis, liver disease, and peripheral vascular disease were reported conditions by 11 (8.5%), 10 (7.7%), 9 (6.9%), 8 (6.2%), and 7 (5.4%) of them, respectively. There were relatively uncommon medical conditions such as depression at 3 (2.3%), psychoses at 2 (1.5%), rheumatoid arthritis at 2 (1.5%), metastatic cancer at 1 (0.8%), and peptic ulcer disease at 1 (0.8%). Notably, no participants reported having AIDS, lymphoma, or paralysis (Table 2). 

Substance use: Smoking was reported by 14 (10.8%) participants (Table 2). The remaining 13 (10.0%) were ex-smokers (Table 2). Drug abuse was also low, with 2 (1.5%) participants affected (Table 2). Alcohol consumption was relatively low, with only 1 (0.8%) participant reporting alcohol use (Table 2). 

Reason for readmission 

**Figure 1:**
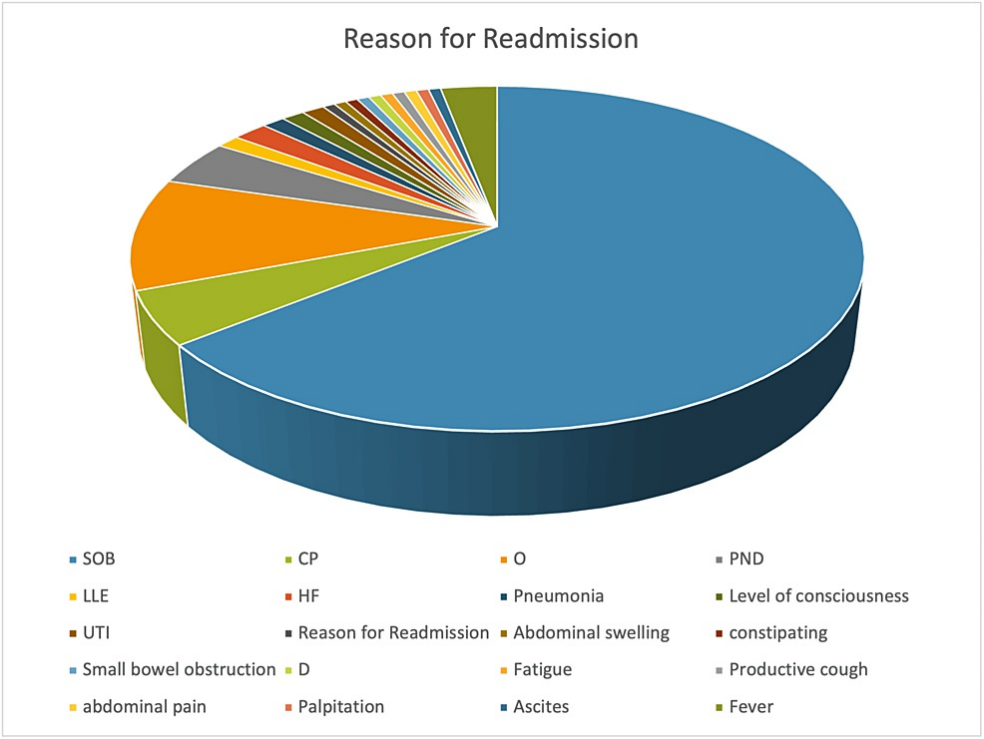
Reasons for readmission among study participants (n=130). The data illustrates the distribution of reasons for readmission among heart failure patients. The data is presented in terms of percentages for reasons of readmission among the total population. SOB, shortness of breath; CP, chest pain, O, orthopnea; PND, paroxysmal nocturnal dyspnea; LLE, lower limb edema; HF, heart failure; UTI, urinary tract infection; D, dementia.

Data in Figure 1 illustrates the distribution of reasons for readmission among HF patients. The commonest reported manifestation accompanying HF patients was shortness of breath (SOB), accounting for (80%) of readmissions; orthopnea and chest pain (CP) represented (13.1%) and (6.2%) of readmissions, respectively. Paroxysmal nocturnal dyspnea (PND) was reported at 5.4%. Lower limb edema (LLE), HF, fever, pneumonia, altered level of consciousness, and urinary tract infections contributed to the remaining (13.8%) of readmissions. 

Analysis of reasons for readmission by age group

A detailed analysis of readmission rates across different age groups for each reason for readmission was conducted to identify any age-related trends (Table 3).

**Table 3:** Distribution of reasons for readmission among different age groups. This table provides information on various reasons for readmission and their prevalence among different age groups. The data is presented in terms of the number of individuals affected and the percentage of the total population with their p-value (consider significant if the P value is less than <0.05).

		Age						Total (N=130)	P value
		30 -40	41-50	51 -60	61 -70	71- 80	81 -90		
Reason for readmission	Chest Pain	0	0	1	1	0	0	2	0.895
		0.0%	0.0%	0.8%	0.8%	0.0%	0.0%	1.5%	
	Orthopnea	1	3	3	6	2	2	17	0.822
		0.8%	2.3%	2.3%	4.6%	1.5%	1.5%	13.1%	
	Paroxysmal nocturnal dyspnea	1	1	4	1	0	0	7	0.127
		0.8%	0.8%	3.1%	0.8%	0.0%	0.0%	5.4%	
	Shortness of breath	2	11	26	30	18	17	104	0.383
		1.5%	8.5%	20.0%	23.1%	13.8%	13.1%	80.0%	

Chest pain (CP): No cases of readmissions were documented in the age groups 30-40, 41-50, 71-80, and 81-90. Conversely, a singular readmission, constituting 0.8%, was observed in both the 51-60 and 61-70 age groups. The associated p-value for this category was 0.626, which signifies a lack of statistical significance concerning age-related differences in readmissions associated with CP (Table 3).

Orthopnea: The distribution of readmissions for edema varied across different age groups. The highest number of readmissions (6) was observed in the age group 61-70, followed by three readmissions in the age group 51-60, constituting proportions of 4.6% and 2.3%, respectively. Conversely, the lowest number of readmissions (one) was reported in the age group 30-40, accounting for 0.8%, and three cases in the age group 41-50, representing 2.3%. In total, the orthopnea category contributed to 13.1% (17) of the overall readmissions (Table 3).

Paroxysmal nocturnal dyspnea (PND): The distribution of readmissions within the PND category exhibited comparatively lower counts in contrast to other categories. The age group with the highest number of readmissions was 51-60, comprising four cases or 3.1%, while the lowest numbers (one case each) were reported in the age groups 30-40, 41-50, and 61-70, each representing 0.8%. The combined impact of the PND category on overall readmissions constituted 5.4% (7) (Table 3).

Shortness of breath (SOB): The SOB category recorded the highest number of readmissions across all age groups. The highest number of readmissions (30) was observed in the age group 61-70, followed by 26 readmissions in the 51-60 age group, constituting 23.1% and 20%, respectively. Conversely, the lowest number of readmissions (two) occurred in the 30-40 age group, representing 1.5%. The SOB category contributed substantially to the overall readmissions, accounting for 80% (104) highlighting a significant association between SOB and readmissions (Table 3).

Interpretations: The findings suggest that age-related differences exist in the distribution of readmission rates for certain reasons for readmission, particularly orthopnea and PND. Further research is warranted to investigate the underlying causes of these age-related variations and to develop targeted interventions for specific age groups.

Readmission causes and gender differences

An analysis of readmission causes revealed that the predominant factor was SOB, constituting 80% (104) of the total readmissions. Other contributing factors included orthopnea at 13.1% (17), CP at 1.5% (2), and PND at 5.4% (7). Notably, gender-based comparisons demonstrated no statistically significant variations in readmission rates between male and female patients concerning CP and PND. Both genders exhibited comparable readmission rates for these specific reasons. However, a conspicuous gender disparity emerged in readmission rates related to SOB. Female patients accounted for 31.5% (41) of readmissions attributed to SOB, whereas male patients constituted 48.5% (63). This observed gender discrepancy prompts the need for further investigation to identify potential underlying factors contributing to this difference (Table 4).

**Table 4:** Differences in readmission rates based on gender and reason for readmission. This table provides information on various reasons for readmission and their prevalence among different genders. The data is presented in terms of the number of individuals affected and the percentage of the total population with their p-value (consider significant if the P value is less than <0.05).

		Gender		Total (N=130)	P value
		Male	Female		
Reason for readmission	Chest pain	1	1	2	0.753
		0.8%	0.8%	1.5%	
	Orthopnea	12	5	17	0.374
		9.2%	3.8%	13.1%	
	Paroxysmal nocturnal dyspnea	3	4	7	0.318
		2.3%	3.1%	5.4%	
	Shortness of breath	63	41	104	0.928
		48.5%	31.5%	80.0%	

Interpretation: The findings highlight the overwhelming dominance of SOB as the primary cause of readmission among heart failure patients, emphasizing the crucial role of effective symptom management and patient education. Additionally, the gender disparity observed in SOB-related readmissions suggests the need for exploration into potential underlying causes and gender-specific interventions to address this issue.

## Discussion

HF patients have experienced improved outcomes due to advancements in medical care [6]. However, readmission rates remain a significant concern. Research suggests that approximately 25% of HF patients are readmitted to the hospital within 90 days of discharge [6].

Non-adherence to medication regimens and lifestyle modifications are significant contributors to readmission rates among HF patients. Non-compliance with medication instructions and a failure to adopt recommended lifestyle changes, such as increased physical activity and reduced salt intake, can lead to exacerbations of symptoms and necessitate readmission. Comorbid conditions, including diabetes, chronic obstructive pulmonary disease, and renal failure, further complicate the management of HF and elevate the likelihood of readmission [10].

A substantial proportion of readmissions were attributable to SOB, underscoring the need to thoroughly evaluate and manage this symptom. Chest pain and edema were also common reasons for readmission, warranting further investigation and targeted interventions. PND, lower limb edema issues, and HF were additional factors contributing to readmission. These conditions demand close monitoring and tailored treatment plans to minimize readmission rates. The presence of fever, pneumonia, altered level of consciousness, and urinary tract infection as reasons for readmission underscores the importance of infection prevention measures and timely medical intervention. While each of the remaining reasons for readmission, including abdominal swelling, constipation, small bowel obstruction, fatigue, productive cough, abdominal pain, palpitation, ascites, and fever, accounted for less than 1% of cases, they still warrant attention and appropriate management to prevent recurrent hospitalizations.

Previous research has indicated that approximately 20% of Medicare beneficiaries experience readmission within 30 days of initial admission, highlighting the prevalence of this issue [4,11]. A study further revealed that 28% of patients are readmitted within 90 days, underscoring the high likelihood of readmission during this period. Transitional interventions may hold promise in reducing readmissions, considering the early occurrence of most readmission events. Commonly employed readmission risk models, such as the hospital-level Centers for Medicare and Medicaid Services (CMS) model, utilize retrospective administrative data to identify relevant variables [12,13]. While pre-admission risk factors are crucial for hospital accountability regarding readmission rates, post-discharge care planning should also incorporate in-hospital factors, such as worsening renal function or HF, into risk assessment models. Certain factors have demonstrated strong predictive power, including HF complications during index hospitalization [14]. Notably, the association between percutaneous coronary intervention and reduced readmission risk suggests that the procedure serves as a patient status indicator rather than a protective factor [15].

Non-compliance with medical regimens and its impact on readmission rates in HF patients

Several studies have demonstrated a strong association between non-adherence to prescribed medical regimens and an increased risk of readmission among patients with HF [16]. This underscores the critical role of patient behavior in shaping health outcomes. One study revealed a significantly higher probability of readmission within 90 days of initial discharge for patients who were non-compliant with their medications and follow-up appointments [16]. This highlights the importance for healthcare providers to effectively address and support patient compliance, thereby reducing readmission rates and improving overall outcomes for individuals with HF [16].

BMI and its correlation with readmission risk in HF patients

Previous research has also established a correlation between body mass index (BMI) and the likelihood of readmission in HF patients [17]. This suggests that maintaining a healthy weight may play a crucial role in mitigating readmission risk for individuals with HF. Healthcare providers should consider this factor when developing treatment plans for HF patients and provide support and resources for weight management [17]. Additionally, educating and counseling patients on the importance of maintaining a healthy weight could prove beneficial in reducing their risk of readmission [17].

By enhancing patient compliance and addressing weight management for improved HF outcomes, by effectively addressing non-compliance and promoting weight management strategies, healthcare providers can significantly contribute to improved health outcomes for patients with HF. This holistic approach can reduce readmission rates, improve quality of life, and potentially lower healthcare costs

Diabetes mellitus and its association with readmission risk in HF patients

Previous studies have provided valuable insights into the relationship between diabetes mellitus and readmission risk in patients with HF. These findings hold significant implications for healthcare professionals, researchers, and policymakers in their efforts to optimize outcomes for individuals with HF and diabetes mellitus [18]. A key takeaway from these studies is the strong association between diabetes mellitus and an increased risk of readmission within 90 days of discharge for HF patients [18]. This highlights the importance of comprehensive management strategies for diabetes mellitus in HF patients, including glycemic control, blood pressure regulation, and lifestyle modifications [18].

Hypertension (HTN) as a risk factor for HF readmission

Numerous studies have consistently demonstrated that hypertension (HTN) is a significant risk factor for readmission in HF patients within a relatively short timeframe [19]. A key finding from these studies is the strong association between uncontrolled HTN and increased risk of HF readmission. Patients with uncontrolled HTN are more likely to experience worsening of their HF symptoms, leading to higher rates of readmission [19]. This underscores the importance of effective blood pressure control in HF patients to mitigate readmission risk [19]. The impact of HTN on HF readmission extends beyond just the presence of the condition. Studies have shown that the severity of HTN, as indicated by higher blood pressure levels, is also correlated with an elevated risk of HF readmission [19]. This emphasizes the need for comprehensive management of HTN, including regular monitoring and aggressive treatment, to reduce the risk of readmission in HF patients [19]. In addition to the direct effects of HTN on HF readmission, previous studies have also identified potential mechanisms underlying this relationship. HTN is known to contribute to the development and progression of HF through its detrimental effects on the heart and vasculature [19]. This can lead to complications such as left ventricular hypertrophy, diastolic dysfunction, and myocardial ischemia, all of which can increase the likelihood of HF readmission [19].

Arrhythmia as a risk factor for HF readmission

Studies have consistently demonstrated a strong association between arrhythmia and an increased risk of readmission in HF patients within a relatively short timeframe [20]. These findings have significant implications for the management and care of HF patients, emphasizing the need for close monitoring and targeted interventions for those with arrhythmias [20]. Arrhythmias, particularly atrial fibrillation (AF), are common comorbidities in HF patients and have been shown to independently increase the risk of readmission [20]. AF can lead to hemodynamic instability, reduced cardiac output, and increased thromboembolic risk, all of which can contribute to worsening HF symptoms and the need for hospitalization [20]. The findings on the association between arrhythmias and HF readmission underscore the importance of routine arrhythmia screening and management in HF patients [20]. Early detection and treatment of arrhythmias can significantly reduce the risk of readmission and improve overall outcomes for HF patients [20].

Anemia and its impact on HF readmission

Previous studies have demonstrated a strong association between anemia and an increased risk of readmission in HF patients within 90 days [21]. Anemia is believed to exacerbate the symptoms of HF, leading to more frequent hospitalizations [21]. The findings on anemia and HF readmission highlight the importance of addressing anemia in HF patients to reduce the risk of readmission [21]. Healthcare providers should routinely monitor hemoglobin levels in HF patients and implement appropriate interventions to correct anemia [21].

Limitation 

The absence of comprehensive medical record details introduces potential bias, underscoring the imperative for methodological precision. This limitation emphasizes the necessity for meticulous approaches in addressing and ameliorating the influence of missing information to uphold the integrity of the study. Additionally, the monocentric nature of the study, confined to a solitary health facility, underscores the necessity for cautious generalization of findings to broader populations. These limitations accentuate the need for future research endeavors to encompass diverse healthcare settings and foster a more comprehensive understanding of the multifaceted dynamics inherent in our subject matter.

## Conclusions

Heart failure (HF) patients continue to face a substantial risk of readmission, highlighting the need for a multifaceted approach to care. Non-adherence to treatment regimens, underlying comorbidities, inadequate discharge planning, and social determinants of health (SDOH) all contribute to this high readmission rate. Additionally, gender disparities in readmission risk exist, with males tending to exhibit a higher prevalence of shortness of breath and subsequent readmission. To effectively reduce readmission rates, healthcare providers must adopt a comprehensive management strategy that addresses these contributing factors. Enhancing patient adherence to medication and lifestyle modifications is crucial, as is aggressive management of comorbid conditions. Additionally, meticulous discharge planning should ensure patients have the necessary education and resources to effectively self-manage their condition. Addressing SDOH factors, such as poverty, social isolation, and limited access to healthcare, is also essential to optimize patient outcomes. By implementing these comprehensive strategies, we can significantly reduce readmission rates for HF patients, improve their quality of life, and mitigate the burden on the healthcare system. Finally, we are strongly advised to prioritize substantial risk factors, including medication-related complaints, diabetes, and hypertension, as well as infections such as pneumonia and urinary tract infections. Furthermore, we advocate for future research endeavors to adopt a multicenter approach and employ meta-analysis methodologies to enhance the robustness of results.

## References

[1] Sanches Machado d'Almeida K, Ronchi Spillere S, Zuchinali P, Corrêa Souza G (2018). Mediterranean diet and other dietary patterns in primary prevention of heart failure and changes in cardiac function markers: a systematic review. Nutrients.

[2] Aldihan DA, Alghafees MA, Alharbi RO, Allahidan RS, AlOmar RH, Alenazi AF, Suliman IF (20211). Readmission rates of heart failure and their associated risk factors in a tertiary academic medical city in Riyadh, Saudi Arabia. J Nat Sci Med.

[3] Hines AL, Barrett ML, Jiang HJ, Steiner CA (2006). Conditions with the largest number of adult hospital readmissions by Payer, 2011. In: Healthcare cost and utilization project (HCUP) statistical briefs.

[4] Dharmarajan K, Hsieh AF, Lin Z (2013). Diagnoses and timing of 30-day readmissions after hospitalisation for heart failure, acute myocardial infarction, or pneumonia. Jama.

[5] Ziaeian B, Fonarow GC (2016). The prevention of hospital readmissions in heart failure. Prog Cardiovasc Dis.

[6] Harmon D, Rathousky J, Choudhry F (2020). Readmission risk factors and heart failure with preserved ejection fraction. J Am Osteopath Assoc.

[7] Chamberlain RS, Sond J, Mahendraraj K, Lau CS, Siracuse BL (2018). Determining 30-day readmission risk for heart failure patients: the Readmission After Heart Failure scale. Int J Gen Med.

[8] Sommerfeld AJ, Althouse AD, Prince J, Hickey GW (2016). Obstructive sleep apnea is associated with increased readmissions in CHF patients. Journal of Cardiac Failure.

[9] Tsuchihashi M, Tsutsui H, Kodama K (2001). Medical and socioenvironmental predictors of hospital readmission in patients with congestive heart failure. Am Heart J.

[10] Al-Tamimi MA, Gillani SW, Abd Alhakam ME, Sam KG (2021). Factors associated with hospital readmission of heart failure patients. Front Pharmacol.

[11] Brown JR, Chang CH, Zhou W, MacKenzie TA, Malenka DJ, Goodman DC (2014). Health system characteristics and rates of readmission after acute myocardial infarction in the United States. J Am Heart Assoc.

[12] Kini V, Peterson PN, Spertus JA (2018). Clinical model to predict 90-day risk of readmission after acute myocardial infarction. Circ Cardiovasc Qual Outcomes.

[13] Kansagara D, Englander H, Salanitro A, Kagen D, Theobald C, Freeman M, Kripalani S (2011). Risk prediction models for hospital readmission: a systematic review. JAMA.

[14] Hess CN, Hellkamp AS, Roe MT (2016). Outcomes according to cardiac catheterization referral and clopidogrel use among medicare patients with non-st-segment elevation myocardial infarction discharged without in-hospital revascularization. J Am Heart Assoc.

[15] Pencina MJ, D'Agostino RB Sr (2015). Evaluating discrimination of risk prediction models: the C statistic. JAMA.

[16] Sharma RK, Dhakarwal P, Violago J, Bhasin R, Ghobrial I (2012). Tackling the readmission epidemic: a resident teaching service perspective. J Community Hosp Intern Med Perspect.

[17] Cox ZL, Lai P, Lewis CM, Lindenfeld J (2020). Body mass index and all-cause readmissions following acute heart failure hospitalization. Int J Obes (Lond).

[18] Thyagaturu HS, Bolton AR, Li S, Kumar A, Shah KR, Katz D (2021). Effect of diabetes mellitus on 30 and 90-day readmissions of patients with heart failure. Am J Cardiol.

[19] Grassi G, Seravalle G, Quarti-Trevano F, Dell'Oro R, Bolla G, Mancia G (2003). Effects of hypertension and obesity on the sympathetic activation of heart failure patients. Hypertension.

[20] McKinley D, Moye-Dickerson P, Davis S, Akil A (2019). Impact of a pharmacist-led intervention on 30-day readmission and assessment of factors predictive of readmission in African American men with heart failure. Am J Mens Health.

[21] Siddiqui SW, Ashok T, Patni N, Fatima M, Lamis A, Anne KK (2022). Anemia and heart failure: a narrative review. Cureus.

